# Musical Attention Control Training for Psychotic Psychiatric Patients: An Experimental Pilot Study in a Forensic Psychiatric Hospital

**DOI:** 10.3389/fnins.2019.00570

**Published:** 2019-06-07

**Authors:** R. van Alphen, G. J. J. M. Stams, L. Hakvoort

**Affiliations:** ^1^Inforsa, Forensic Psychiatric Hospital, Amsterdam, Netherlands; ^2^Social and Behavioral Sciences, University of Amsterdam, Amsterdam, Netherlands; ^3^Department of Music Therapy, ArtEZ University of the Arts, Enschede, Netherlands

**Keywords:** musical attention control training, forensic psychiatry, psychosis, psychiatric patients, randomized controlled trial, sustained, selective, alternating

## Abstract

Poor attention skills constitute a major problem for psychiatric patients with psychotic symptoms, and increase their chances of treatment drop-out. This study investigated possible benefits of musical attention control training (MACT). To examine the effect of MACT on attention skills of psychiatric patients with psychotic features a randomized controlled trial (RCT) was conducted in a forensic psychiatric clinic. Participants (*N* = 35, age *M* = 34.7, 69% male) were pair matched (on age, gender, and educational level), and randomly assigned to an experimental and control group. The experimental group received a 30-min MACT training once a week over 6 weeks’ time, whereas the controls received treatment as usual without attention training. Single blind pre- and post-neuropsychological assessments were performed to measure different attention levels. The experimental MACT group outperformed the control group in selective, sustained and alternating attention. In addition, overall attendance of MACT participants was high (87.1%). This result suggests that in this experimental pilot study MACT was effective for attention skills of psychiatric patients with psychotic features. To obtain larger intervention effects additional research is necessary, with a larger sample and a more specific MACT intervention.

## Introduction

About 0.4% of the world population suffers from psychotic episodes or schizophrenia (McGrath et al., 2004). Most of the patients receive regular treatment (Vancampfort et al., 2016). However, 26.7% of these patients cannot complete treatment (Vancampfort et al., 2016). Some patients might become a possible threat to themselves or other people (Pompili et al., 2007; Large and Nielssen, 2011). Yearly roughly 30 thousand of them are placed under a legal order in secure psychiatric setting in the Netherlands (Eshuis and Diephuis, 2017; Dienst Justitiële Inrichtingen, 2018) due to conviction for criminal offenses or severe violent threats to themselves or others. The number of patients in these settings has doubled over the last 10 years (Broer et al., 2015). Treatment costs differ from aaa402 to aaa528 per patient per day. Treatment can take years and can cost up to 1.5 million euro per patient (Nagtegaal et al., 2016). These costs are covered because of the positive effects of forensic treatment. Of these patients, 20.7% relapse into a severe criminal offense compared to the three times higher 64% of largely untreated adult delinquents, who only served a prison sentence (Boonmann et al., 2015). Treatment success is often measured as reduction of relapses. Effective treatment is essential for rehabilitation of patients, and reduction of costs.

Patients with psychotic features in forensic psychiatric settings have many cognitive problems (Heinrichs and Zakzanis, 1998; Kavanagh et al., 2010; Meyer et al., 2014). Often judgment functions fail, patients are easily distracted, and they show neurocognitive deficits, such as sustained attention problems, impulsivity and executive dysfunction. One could be motivated to partake in treatment, but when constantly distracted, it is hard to learn new skills or gain new insights. In search of predictors and moderators of treatment success, research suggests that the inability to focus and sustain attention are neurobiological predictors of negative treatment outcomes (Cornet et al., 2015). The worse the attention span of a patient the more difficulties are denoted in maintaining and completing treatment programs.

Attention span and related problems evolve around a person’s capacity to absorb and apply information. In daily life, we are (over)exposed to information. This information can be auditory-, tactile-, visual stimuli (external) as well as thoughts, sensations, emotions, and memories (internal). It is estimated that our human system unconsciously can process 11,2 million bits of information every second (Dijksterhuis and Nordgren, 2006). In contrast, most people are aware of only 45 bits per second; 200.000 times less. The selection mechanism that filters this information is called attention (Kessels et al., 2012).

Humans have only a limited attention capacity (Dijksterhuis and Nordgren, 2006), which even slims when alternating between multiple stimuli. When learning a new task, like playing a guitar, our attention is heavily occupied. Processing all the new information takes time, and it is extremely difficult to focus attention on other tasks in that situation. After repetition and training, these processes proceed more automatically, and therefore demand less attention capacity (Shiffrin and Schneider, 1977). After many hours of rehearsal, we can even sing and play guitar at the same moment. Attention is an essential skill to be able to reason, read, learn and communicate. Attention helps us to cope with and solve problems (Petersen and Posner, 2012).

Cognitive function disorders appear to be one of the core characteristics of schizophrenia and psychotic episodes and are observable to varying degrees within the areas of attention, memory, language and executive functions (Kavanagh et al., 2010). Cornet et al. (2015) have suggested that there is a significant treatment attrition of psychiatric patients with poor attention skills. They found that patients who are the most in need of treatment benefit the least of it, probably due to inadequate attention skills. However, psychological treatment that targets attention skills is a fairly uncovered area of research.

Posner distinguishes four different levels of attention (Posner, 2016). Focused attention, selective attention, sustained attention, and alternating attention. Since the capacity of a human information selection mechanism is limited, attention needs to be focused and selective. *Focused attention* is the ability to direct attention to stimuli. *Selective attention* is the ability to select one stimulus over another. It is the ability to avoid distractions when focused (Kessels et al., 2012). This can be controlled as a bottom-up process. For example, when involuntary stimuli, like a claxon in traffic, draws attention. Top-down attention implies the capacity to actively focus on stimuli of interest, such as reading this article. Thirdly, certain tasks require one to *sustain* or hold attention over time. One needs to be alert when performing vigilance tasks with a low event rate, like driving a car for hours on a highway. *Alternating attention* is the ability to switch in a sequence between one thing and another. The tempo of alternating attention can vary from calm to rapidly (sometimes referred to as divided attention). When investigating training effects on attention skills, it is important to distinguish between these levels of attention.

If one wants to train attention skills one should focus on the neuropsychological aspects of attention (Silverstein et al., 2001). Research suggests that listening to music stimulates brain plasticity (Pantev and Herzholz, 2011) and leads to activations in multiple brain areas, such as auditory mechanisms, attention, memory storage and retrieval, and sensory–motor integration (Zatorre, 2005). Making music involves the brainstem, limbic systems and frontal lobes (Alluri et al., 2011; Meyer et al., 2014), stimulates the visual- and motor cortex (Collins, 2013), and leads to volume- and activity increases of the corpus callosum (Schlaug et al., 1995; Steele et al., 2013). A growing number of studies suggest that many brain areas involved in attention processes are activated by music (Schmithorst and Holland, 2003; Thaut, 2010; Pantev and Herholz, 2011; Strait and Kraus, 2011; Miendlarzewska and Trost, 2013; Thaut and Gardiner, 2014; Mansouri et al., 2017). An effect study on music therapy and attention showed improvement in learning skills of patients diagnosed with schizophrenia (Ceccato et al., 2006). However, the music therapy interventions in this study were not well-defined. Building on knowledge of three different attention studies (Klein and Jones, 1996; Ceccato et al., 2006; Wolfe and Noguchi, 2009; Thaut and Gardiner, 2014) developed the musical attention control training (MACT). MACT is a protocolled neurological music therapy (NMT) technique targeting music brain mechanisms in structured music making or listening exercises to optimize attention processes. These exercises consist of pre-composed or improvised music. The musical elements cue different musical responses to practice attention skills (Thaut, 2005), such as brainstem reflex, entrainment, and contagion (Juslin, 2019).

A literature review only provided three studies with extreme small sample sizes on the effects of MACT on attention. Abrahams and Van Dooren compared MACT to non-standardized music therapy (NSMT) and treatment as usual (TAU) on attention skills of juveniles in a secure residential facility (*N* = 6) (Abrahams and Van Dooren, 2018). The participants were randomly allocated to one of various treatment groups. Participants (age: *M* = 16,5, *SD* = 0.5) were diagnosed with Attention Deficit (Hyperactivity) Disorder, Oppositional Defiant Disorder and/or Conduct Disorder. Participants were given a six-week training of 45 min weekly. To measure attention the trail making test A and B (TMT) and the Digit Span, forward and backward, were used. The two participants in the MACT group showed a significant increase in selective, sustained and divided attention areas compared to the others.

Pasiali, LaGasse, and Penndid investigated attention skill training in a single group pre- /post-test study. Their aim was to find an effect of MACT on attention skills of adolescents with neurodevelopmental delays (Pasiali et al., 2014). Participants (*N* = 9) aged 13–20 were diagnosed with minor, mild or severe symptoms of autism. Participants were given eight 45-min sessions spread over 6 weeks’ time. Attention was measured with the test of everyday attention for children (TEA-ch). Participants showed significant improvements on selective attention and divided attention.

In an unpublished study, Roefs conducted a randomized controlled trial (RCT) with forensic psychiatric male adults suffering from schizophrenia (Roefs, 2015). He compared music therapy combined with MACT (CM) with improvisation music therapy (IM) for (*N* = 14) forensic psychiatric patients diagnosed with schizophrenia (ranging in age from 18 to 65). Here too, the TMT A and B, the digit span forward and backward in addition to the parrot digits and letters were used to measure attention. Participants in the CM demonstrated improvement in focused, sustained and alternating attention.

Even though all studies had their limitations, the current literature review led to the hypothesis that MACT could have a positive effect on the development of focused and selective attention as well as sustained and alternating attention skills of psychiatric patients in forensic psychiatry. By conducting a randomized controlled trial, the present study aims to find the effect of MACT on attention skills within a group of adult psychiatric patients with psychotic features who stay in a secure psychiatric setting. The main question is: “What are the effects of MACT on focused, selective, sustained, and alternating attention in psychiatric patients with psychotic features in secure residential care?”

## Materials and Methods

### Design

**FIGURE 1:**
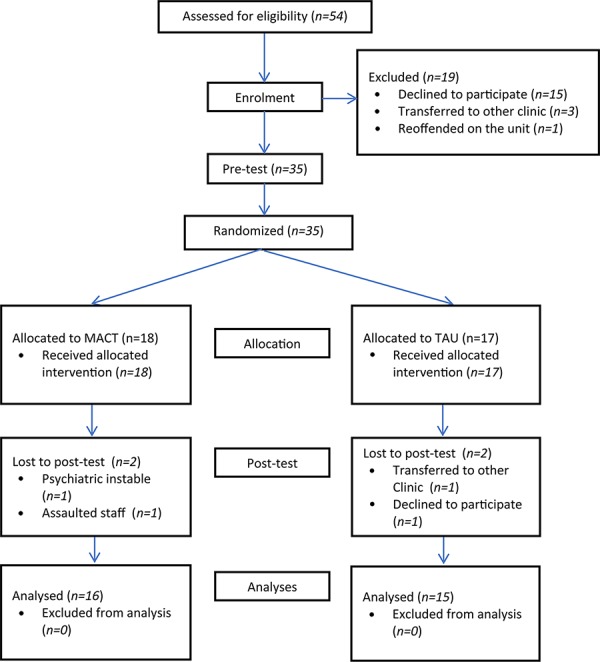
Flow chart of participants.

This study employed a single blind randomized controlled pre-/post-test design and was approved by the Medical Ethics Committee of the University of Amsterdam (registration number 2018-CDE-2968). Julious (2005) state when conducting a pilot study, a sample size of 12 participants per group is a rule of thumb. Prior to the randomization all participants participated in a pre-test assessment. To create a comparable control and experimental group patients were matched (on age, gender and educational level) before being allocated to the experimental group (receiving the MACT training in addition to treatment of usual (TAU)) or the control group (only receiving TAU). The control group was placed on a waiting list for MACT training. Participants of both groups were subjected to a post-test assessment immediately after the sixth session or sixth week (see **Figure 1**). After the post-test assessment the control group was offered to enroll in the MACT training.

### Participants

In total 54 patients were found eligible for the MACT treatment program. Inclusion criteria were: adult psychiatric patients (18+ years) with psychotic features in a forensic clinical setting who had severe attention problems, who mastered the Dutch language on at least a basic level. Patients had to be able to leave the unit independently to visit the music therapy room. Exclusion criteria were: acute crisis, florid psychosis or patients who were in a coercive program (i.e., having no permission to leave the unit). There was no exclusion for additional psychiatric disorders, age, gender or educational level.

After informing all eligible patients about the study a total of 15 patients refused to participate. Four left the study before allocation. Three of them were transferred to another psychiatric institution. One committed arson the day before the research started and was deemed to be unsuited to leave the unit. This resulted in a sample of 35 participants (age *M* = 34.7, *SD* = 9.6, 69% male) (see **Figure 1** for details). All participants were patients of a secure psychiatric clinic and stayed in one of three units; short-term coercive psychiatry, long-stay secure psychiatry or forensic psychiatry. A total of 35 participants completed the pre-test, compared to 31 who completed the post-test. There were two dropouts in each group.

### Procedure

Patients with severe attention problems were referred to participate in the study by their psychiatrists and psychologists. All patients who were enlisted for the treatment were personally invited by one of the three neurologic music therapists who would offer the MACT. The patients received information about the MACT, the research design, the voluntary participation, the possibility to withdraw, and were asked if they had attention problems. Later on, patients who wanted to participate were required to sign an informed consent form.

All patients did a pre-test attention skill assessment in the same week (week 19 of 2018). Attention skills have been shown to differ across age, gender and educational level, and can influence the outcome of treatment (Bates and Lemay, 2004; Tombaugh, 2004). For that reason, prior to randomization, patients were matched on age, gender, psychiatric unit and educational level. Since a third of the patients did not finish high school, the educational level was set on the highest level of education they had attended. Although the sample size of the experimental and control group exceeded the rule of thumb for a pilot study (Julious, 2005), the sample size of this RCT was considered to be too small to secure equivalence of the experimental and control group. Participants were therefore pair-matched after the pre-test assessment. One of the researches entered the patients’ variables onto a list and matched similar patients (for example a male patient, born in 1982 who did not finish his lower secondary education was matched with a male patient, born in 1984 who also did not finish his lower secondary education). By flipping a coin, the group was allocated to the conditions. A total of 18 patients (age *M* = 34.4*, SD* = 9.4) were assigned to the experimental group and 17 (age *M* = 35.2, *SD* = 10.1) to the control group (see **Table 1** for Participants’ Characteristics). The experimental and control group turned out to be similar on age (*p* = 0.768), gender (*p* = 0.810) and educational level (*p* = 0.826). Participants were not matched on psychiatric disorders, but visual inspection of **Table 1** suggest that the distribution of psychiatric disorders were similar. The neuropsychological tests were carried out under supervision of independently trained professionals. The data were anonymized and handed over to the researchers. Participants and assessors only learned who would participate in the experimental or control group after every participant had completed the pre-test.

**Table 1:** Participants’ Characteristics.

	Experimental (*N* = 18)	Control (*N* = 17)
Male/Female	12 (67%)/6 (33%)	12 (71%)/5 (29%)
Age	*M* = 34,39 SD = 9,39	*M* = 35,12 SD = 10,08
**Level of education**		
No education	1 (6%)	0 (0%)
Primary ed. not completed	0 (0%)	1 (6%)
Primary ed. completed	1 (6%)	1 (6%)
Middle school not completed	1 (6%)	3 (18%)
Middle school completed	8 (44%)	4 (24%)
High school not completed	2 (11%)	1 (6%)
High school completed	0 (0%)	2 (12%)
College not completed	3 (17%)	2 (12%)
College completed Low	2 (11%)	3 (18%)
**Psychiatric disorders**		
Psychotic disorder	12 (67%)	10 (59%)
Autism	4 (22%)	2 (12%)
Bipolar	0 (0%)	2 (12%)
Drugs abuse	10 (56%)	11 (65%)
BPS	3 (17%)	4 (24%)
PTSD	3 (17%)	1 (6%)
Eating disorder	3 (17%)	2 (12%)
OCD	1 (6%)	0 (0%)
Anxiety	0 (0%)	2 (12%)
Depression	1 (6%)	0 (0%)
Received NS-music therapy	14 (78%)	14 (82%)
**Clinic**		
FPK	8 (44%)	7 (41%)
LIZ	6 (33%)	7 (41%)
KIB	4 (22%)	3 (18%)


*BPS, borderline personality disorder; PTSD, post-traumatic stress disorder, OCD, obsessive compulsive disorder; NS-Music therapy, non-standardized music therapy; FPK, forensische psychiatrische kliniek (forensic psychiatric clinic); LIZ, langdurige intensieve zorg (long-term intensive care); KIB, kortdurende intensieve behandeling (short-term intensive treatment)*.

Participants in the experimental group followed the MACT, which started a week after the pre-test. Participants in the control group attended their regular treatment program and received no additional intervention. They started with MACT after the post-test. A total of 69% of the participants had received non-standardized music therapy (NSMT) prior to this study equally distributed over both groups. Therapy goals aimed to introduce music therapy, observation, diminishing negative symptoms of psychosis, increase interaction skills, regulate emotions or increase self-esteem. However, none of the participants received MACT or other neurologic music therapy techniques.

The d2 cancelation Test (Brickenkamp and Zillmer, 1998; Brickenkamp, 2007), the Digit Span Forward and Backward (Wechsler, 2012) and the TMT A and B (Tombaugh, 2004) were used to measure the different levels of attention.

### The Intervention: MACT

To allow for replication of the study we will provide the reader with a detailed description of the music therapy intervention as well as the applied standardized assessment tools. Den Heijer (2018) designed a six-week protocol-based (MACT-program) (Thaut and Gardiner, 2014) to meet the needs of psychiatric patients with psychotic features, ensuring that the core of each exercise met the MACT criteria, triggering cognitive areas of attention, neural networks and brain systems and functions (for example, bilateral frontal lobes, brain stem, auditory and visual perception systems). Each session lasted 30 min. The session started with psycho-education about targeted attention processes and defining the proposed musical exercises. Questions patients had, were clarified prior to the start of the musical interventions. The attention skills training was gradually increased in complexity. This kept participants motivated for a longer duration of time. The protocol was designed for a closed group with a maximum of six participants. The program had a build-up. It started with musical exercises for focused and selected attention, gradually going to sustained attention, while ending with alternating attention.

The musical equipment used in the exercises were African drums, small percussion, xylophones, metronome, as well as pre-recorded and pre-composed music. Four groups started with MACT-sessions in the same week. The number of participants in the MACT group varied between two to six participants. The protocol was carried out by three experienced and NMT trained music therapists. To meet the institutional requirements a co-therapist was included in each session.

### Measures

In this study three different tests to assess different levels of attentions were used. First, the d2 cancelation test was used to measure sustained attention. To conduct the d2 test one uses visual selective attention, speed of information processing and sustained attention (Brickenkamp, 2007). The purpose of the test is to distinguish different characters.^1^ Two of the twelve measures of the d2 were used, D2TN and D2CP. D2TN is the total sum of number of characters processed (Bates and Lemay, 2004). The D2CP is the correct number of canceled characters minus the falsely canceled characters, and is considered as a measure of sustained attention. Reliability research done with adults found a good reliability for the D2TN and the D2CP both of *α* = 0.98 (Brickenkamp, 2007).

The second attention test was the Digit Span as a subtest of the WAIS-IV-NL intelligence test (Wechsler, 2012). The digit span forward (DF) task tempts the executive functions and correlates with vigilance (Hale et al., 2002). The DF is used to measure focused attention.^2^ The score of the digit span was calculated by the number of correctly named rows. The digit span backward (DB) requires the use of working memory and executive functioning (De Jong and Das-Smaal, 1995), while selecting and maintaining the information additionally requires attention skills. This requires impulse controls and mental flexibility (Hale et al., 2002). Therefore, this test was used to measure selective attention. This test was chosen because, in contrast to the d2 and the TMT A and B, it uses auditory stimuli. For this test a good internal consistency was found of = 0.91 (Wechsler, 2012).

The TMT A and B, developed by Reitan (Reitan, 1958), was the third measure of sustained attention (Reitan, 1958; Benedict et al., 1996; Sánchez-Cubillo et al., 2009).^3^ This TMT-A task requires mainly visioperceptual abilities (Benedict et al., 1996) and working memory (Sánchez-Cubillo et al., 2009). TMT-B measures alternating attention, since it reflects primarily working memory and secondarily task-switching ability (Benedict et al., 1996),^4^ It requires cognitive alternation, inhibition control, working memory, and attentional shifting (Sánchez-Cubillo et al., 2009). The reliabilities found in clinical groups vary from 0.69 to 0.94 for the TMT-A and 0.66 to 0.86 for the TMT-B (The Goldstein and Watson, 1989).

Beside their validity to assess attention skills, the d2 cancelation test was included to enable comparison with the results of Cornet et al. (2015). The Digit Span and the TMT A and B were used to compare the outcomes of this study with both previously mentioned studies on the MACT (Roefs, 2015; Abrahams and Van Dooren, 2018).

### Analysis

To analyze the data IBM SPSS 23 (George and Mallery, 2016) was used. Mean and standard deviations of variables were calculated. Independent T-tests were used to test whether mean scores of focused and selective attention, sustained attention and alternating attention would increase for the MACT participants compared to the control participants. Effect sizes were computed as Cohens’ *d*, based on means and standard deviations, with a positive sign indicating improvement of the experimental group relative to the control group. Effect sizes around 0.20 are marked as small, effect sizes around 0.50 as medium, effects sizes around 0.80 as large (Cohen, 1988). Because the present research was a pilot study and therefore statistically underpowered, with a small sample size, the *p*-value was set to *p* < 0.10 (one-tailed significance) in order to be able to detect (trend significant) clinically meaningful effects.

## Results

A total of 31 participants completed both the pre- and post-test. Pre- and post-scores of both groups were compared for differences in attention skills.

**FIGURE 2:**
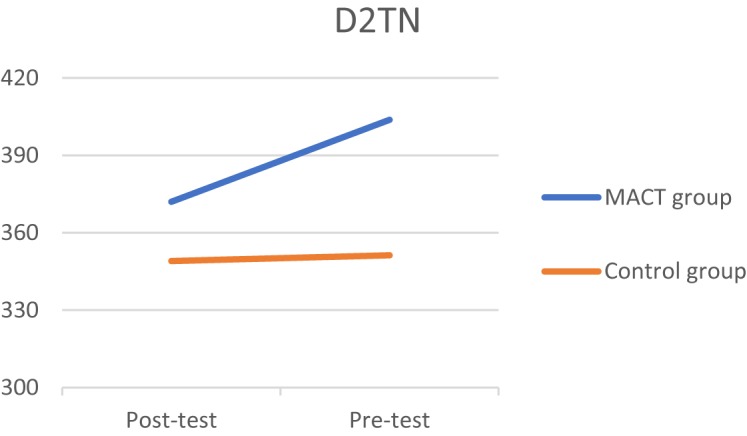
D2TN.

**FIGURE 3:**
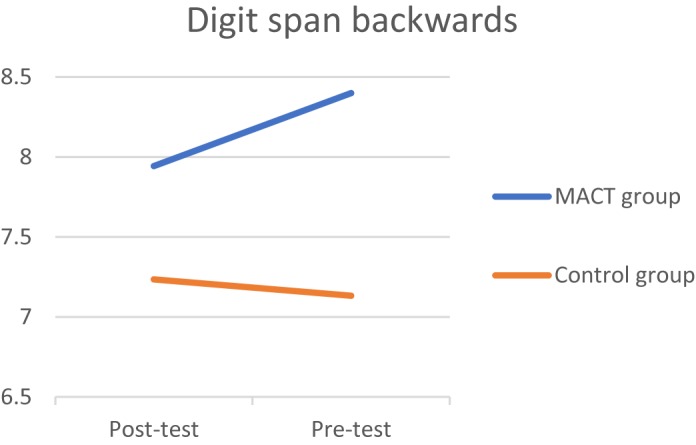
Digit span backward.

**FIGURE 4:**
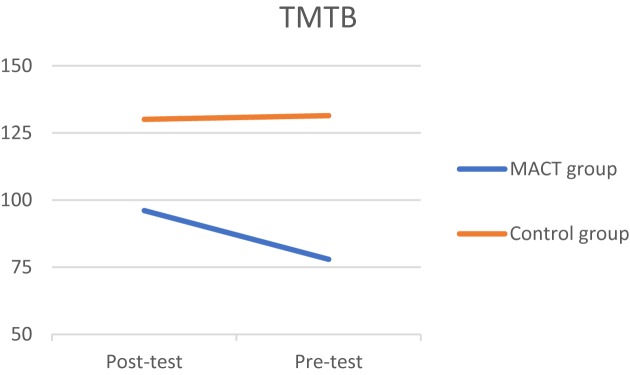
TMTB.

Three significant effects were discovered at *p* < 0.10. Starting with the D2TN, a medium effect size was found (*t* = 1.374*, p* = 0.09, *d* = 0.49), which means that the experimental group had improved in sustained attention (**Figure 2**). A positive effect was found in the digit span backward (*t* = 1.512, *p* = 0.07, *d* = 0.55), improving on selective attention (**Figure 3**). Third, the experimental group took less time to finish the TMTB compared to the control group (*t* = -1.525, *p* = 0.07, *d* = 0.59), which indicates that the experimental group improved in alternating attention (**Figure 4**). These results suggest that the MACT might have a positive effect on attention skills. No effects were found on focused attention.

Despite all measures of precaution in the paired-match randomization, the experimental group scored better on attention skills than did those in the control at pre-test, as shown in **Table 2**.

**Table 2:** Differences pre and post-test.

	*Pre-test*					*Post-test*					*post minus pre-test*
	*N*	*M*	*SD*	*t*	*d*	*N*	*M*	*SD*	*t*	*d*	*d*
D2TN				0.701	0.25				1.374+	0.49	0.24
Test	(*N* = 16)	371.81	89.081			(*N* = 16)	403.88	97.118			
Con	(*N* = 16)	349.31	92.346			(*N* = 15)	351.27	115.803			
D2CP				0.297	0.10				0.792	0.28	0.18
Test	(*N* = 16)	135.94	46.323			(*N* = 16)	148.19	45.809			
Con	(*N* = 16)	131.13	45.425			(*N* = 16)	134.67	49.209			
DF				0.659	0.22				1.013	0.36	0.14
Test	(*N* = 18)	8.50	2.684			(*N* = 16)	8.94	2.265			
Con	(*N* = 17)	7.94	2.331			(*N* = 15)	8.07	2.520			
DB				0.964	0.32				1.512+	0.55	0.22
Test	(*N* = 18)	7.94	2.287			(*N* = 15)	8.40	2.473			
Con	(*N* = 17)	7.24	2.047			(*N* = 15)	7.13	2.100			
TMTA				-1.123	0.38				-0.474	0.17	-0.21
Test	(*N* = 18)	38.22	14.190			(*N* = 16)	34.69	12.333			
Con	(*N* = 17)	44.06	16.528			(*N* = 15)	36.67	10.788			
TMTB				-0.914	0.33				-1.525+	0.59	0.26
Test	(*N* = 16)	96.06	59.013			(*N* = 15)	77.93	40.136			
Con	(*N* = 15)	115.47	59.130			(*N* = 12)	102.92	44.903			


*Differences between pre and post-test MACT in test and control group: means, standard deviations and effect-sizes Test, test group (15 < n < 18); Con, control group (12 < n < 17).+ <0.10; D2TN, d2 cancelation test total number of letter (sustained – focussed – attention); D2CP, d2 cancelation test correct total number of letters (sustained – selective – attention); DF, digital span forward test (focussed attention); DB, digital span backward test (selective attention); TMT A, trail making test A (sustained attention); TMT B, trail making test B (alternating attention)*.

The therapists reported an overall high therapy attendance of 87%. Perry, Bannon and Ini (Perry et al., 1999) showed that the average attendance in therapy is 78%, suggesting that motivation for the MACT program was high.

## Discussion

With regards to the effect of MACT on different levels of attention the experimental group showed better outcomes on selective, sustained and alternating attention compared to the control group, with medium effect sizes at post-test. So, the results indicated that MACT does have a positive effect on three attention levels, selective, sustained and alternating. This suggests that MACT is an effective music therapy program to train attention skills of psychiatric patients with psychotic features in a secure psychiatric facility. The effect of the MACT, improvement on selective, sustained and alternating attention are in line with other studies (Klein and Jones, 1996; Ceccato et al., 2006; Wolfe and Noguchi, 2009; Thaut, 2010; Pasiali et al., 2014; Roefs, 2015; Abrahams and Van Dooren, 2018). No effects were found on focused and specific forms of alternating attention.

This pilot study suggests that an experimental study design testing the effects of MACT on attention skills of psychiatric patients with psychotic features (in a secure psychiatric institution) is feasible, although the program might need modification in order to obtain larger intervention effects. If the results are replicated in future studies, there remain reasons to be careful with implementation. To our knowledge, only one study, carried out by Cornet and others (Cornet et al., 2015), examined the relation between attention skills and treatment outcome, and found that poor attention skills relate to treatment drop out. Adding a new therapy to an existing treatment program brings questions about its additional value in clinical practice that surpasses issues of effectiveness. MACT was added to treatment as usual offered to the three clinics.

Some qualitative date was gathered by asking patients to provide their thoughts and feedback on the program. They expressed their skepticism (“I don’t think only six sessions will increase attention skills”) or had ideas to modify the musical exercises to make them more challenging (for example, making the rhythms more complex, or playing more difficult melodies on the xylophones). It did not prevent the patients from attending the training. Or as one patient put it; “It was nice to do something, otherwise you’re here to waste, but I don’t know if my attention skills have improved.”

Although a single blind pair matched RCT design was used for this study, some limitations must be acknowledged. The sample was too small for valid generalization and did lack statistical power. Although the direction and magnitude of the effects were similar to earlier studies, still a much larger sample is needed to be able to generalize the present study’s findings. This pilot study should be considered as a test of feasibility to examine the effects of MACT on attention of psychiatric patients with psychotic features (in secure psychiatric care). However, the positive trends of the present study warrant further research to test the effects of MACT on different attention processes for this population.

Another limitation of this study is that confounders that could have affected attention skills were not controlled for. For example, methylphenidate is known to boost sustained attention (Dockree et al., 2017), elective compulsory therapy is known for its loss of temporary memory and for disturbing working memory (Datto, 2000), which is a key component of attention (Kessels et al., 2012). However, clients were not screened for those factors. Drug abuse was similarly not controlled for, although 21 participants (60%) had been diagnosed with substance use disorder. Cannabis reduces the function of the attention system, MDMA users are less efficient at focusing, cocaine users show a lack of attention, and heroin users have problems with impulse control and selective processing (Lundqvist, 2005). Thus, future studies should try to incorporate these additional confounding factors in their analysis.

The Hawthorne effect (McCarney et al., 2007; Rapport et al., 2013) cannot be ruled out because the control group did not receive an alternative intervention to control for the effect of getting attention. Similar to the studies conducted by Abrahams and Van Dooren (Abrahams and Van Dooren, 2018) and Roefs (Roefs, 2015), it would be interesting to offer the control group regular music therapy treatment compared to MACT. However, both studies found that control groups receiving another (placebo) form of music therapy did not improve as much on attention skills as did the experimental group receiving MACT.

Finally, although participants in the experimental and control group were paired on similar age, gender, clinical problems and education level, randomization could not prevent that the experimental group showed higher attention skills at pre-test. it should be noticed that the experimental group in general showed better attention scores at pre-test than did the control group, although not significant due to low statistical power. When results were controlled for pre-test differences, effect sizes were small rather than medium. *Post hoc* ANCOVA’s revealed that post-test differences between the experimental and control group were no longer significant after controlling for pre-test differences. So it should be advised to pair on attention skills in addition to the previously named standards.

## Conclusion

Findings of this pilot study suggest that MACT has a positive effect on attention skills in forensic psychiatry for psychiatric patients with psychotic features. The intervention seems to adequately meet the needs of these patients as evident in the low dropout rate, the good response rate, and the willingness to participate in an RCT. This pleads for replication of this study with a larger sample size. It was calculated with G-power (*power of 0.80 and α = 5%*) that a sample of *N* = 102 would be needed given a medium effect size. It would be advised to intensify or adapt the MACT training to find larger effects. Compared to musical training, rehearsing multiple times a week has been shown to produce more improvement in music skills, which promotes brain plasticity (Wan and Schlaug, 2010). The duration of someone’s music training strengthens neural activation (Pantev and Herzholz, 2011). This means MACT might need broader variation of musical exercises, a prolonged duration and a delivery of multiple times each week. One of the participants suggested additional homework exercises. A possible decrease of treatment dropout through attention improvement would even stronger encourage the implementation of MACT within the treatment of (forensic) psychiatric patients with psychotic features. Cost effectiveness, achieved by a shorter average stay in forensic psychiatry or a greater effectiveness of psychiatric treatment, would be an impetus to implement musical attention control interventions within forensic psychiatric facilities.

## Ethics Statement

The study is approved by the Medical Ethics Committee of the University of Amsterdam (registration number 2018-CDE-2968). Each participant was informed about the content of the study and the possibility to withdraw their consent. Each included participant signed a consent form.

## Author Contributions

RvA conceptualized and directed this research, recruited participants, collected pre/post measures and facilitated the necessary conditions for the research, collected and analyzed the data, and wrote the first draft of the manuscript. GS supervised the research process. LH contributed to the conception and design of the study. All authors contributed to manuscript revision, read and approved the submitted version.

## Conflict of Interest Statement

The authors declare that the research was conducted in the absence of any commercial or financial relationships that could be construed as a potential conflict of interest.

## Footnotes

1 On an a4-sheet, 14 lines alternate the letter “d” and “p” with one to four dashes. The letter “d” with a total of two dashes must be marked, the letter “p” and letter “d” with more or less than 2 dashes must be ignored. For each line there are 20 s to indicate the correct characters.

2 In the DF, digit sets are read out loud. The patient has to repeat the numbers. These number sequences build up in length from 2 digits to 9 digits, and stop when the patient makes two errors in a row. In the Digit Span backward (DB), the patients are expected to recall the digits backward (for example, 2-1-7 becomes 7-1-2).

3 In the TMT-A test patients receive a sheet of paper with 25 numbers, which have to be connected with each other, starting at one and ending at 25. The score is the time patients need to end the test without errors.

4 The set-up of the TMT-B test is equal to TMT-A, but now numbers and letters must be connected alternated to each other (for example, 1-A-2-B-3-C-4-D etc.).
